# COPI Is Required for Enterovirus 71 Replication

**DOI:** 10.1371/journal.pone.0038035

**Published:** 2012-05-25

**Authors:** Jianmin Wang, Zhiqiang Wu, Qi Jin

**Affiliations:** State Key Laboratory for Molecular Virology and Genetic Engineering, Institute of Pathogen Biology, Chinese Academy of Medical Sciences and Peking Union Medical College, Beijing, People's Republic of China; Centro de Biología Molecular Severo Ochoa (CSIC-UAM), Spain

## Abstract

Enterovirus 71 (EV71), a member of the *Picornaviridae* family, is found in Asian countries where it causes a wide range of human diseases. No effective therapy is available for the treatment of these infections. Picornaviruses undergo RNA replication in association with membranes of infected cells. COPI and COPII have been shown to be involved in the formation of picornavirus-induced vesicles. Replication of several picornaviruses, including poliovirus and Echovirus 11 (EV11), is dependent on COPI or COPII. Here, we report that COPI, but not COPII, is required for EV71 replication. Replication of EV71 was inhibited by brefeldin A and golgicide A, inhibitors of COPI activity. Furthermore, we found EV71 2C protein interacted with COPI subunits by co-immunoprecipitation and GST pull-down assay, indicating that COPI coatomer might be directed to the viral replication complex through viral 2C protein. Additionally, because the pathway is conserved among different species of enteroviruses, it may represent a novel target for antiviral therapies.

## Introduction

The enteroviruses belong to the family of *Picornaviridae*. Many enteroviruses cause disease in humans, but the infections are generally mild or asymptomatic. However, enteroviruses may also result in serious or even fatal disease. EV71 can lead to serious neurological diseases in infants and children, such as aseptic meningitis, encephalitis, and acute flaccid paralysis and even death [1], [2]. EV71 has a ∼7.4 kb positive-sense, single-stranded RNA genome, which has a single open reading frame encoding a polyprotein flanked by 5′- and 3′-untranslated regions (UTRs). It has a small, viral protein, VPg, linked to the 5′ end of the genomic RNA, which is involved in the initiation of viral RNA genome replication. During infection, Scavenger receptor B2 (SCARB2) and P-selectin glycoprotein ligand-1 (PSGL-1) work as cellular receptors and mediate the attachment of EV71 to cells [3], [4]. After invading into cells, EV71 uses a mechanism of translation initiation that starts with direct binding of the ribosome to internal ribosome entry site (IRES), which lies in the 5′UTR, allowing viral gene expression to occur in a cap-independent manner while host cell translation is shut down [5]. Since an outbreak in Malaysia in 1997 [6], EV71 outbreaks have been frequently observed in Western Pacific Region countries, including in Australia in 1999 [7], [8], Singapore in 2000 [9], Japan in 1997 and 2000 [10], [11], and Taiwan in 1998 [12]. Sporadic epidemic or outbreaks of EV71 infection were also reported after 2000 [13], [14], [15], [16].

Picornaviruses are non-enveloped, positive-strand RNA viruses. All positive-strand RNA viruses undergo RNA replication in association with membranes of infected cells [17]. However, Picornaviruses generally do not use native organelle membranes for replication but induce the formation of novel cytoplasmic vesicular compartments in infected cells [18], [19]. These membranes can be derived from a variety of sources within the host cell, including the endoplasmic reticulum (ER), Golgi apparatus, mitochondria, or from the endolysosomal compartment [20]. These vesicles are the sites of viral RNA replication and are essential for replication. However, the mechanism by which piconaviruses induce this vesicular compartment and direct their RNA replication complex to these specific intracellular membranes, and the cellular components involved in this process, are not well established. The identification of host proteins and pathways used by viruses to complete their life cycles has given us insights into both host-pathogen interactions and provide potential as novel antiviral targets. Coat protein complex I (COPI) and COPII vesicles are essential components of the trafficking machinery cycling between the ER and Golgi [21]. Each complex is a multisubunit complex. COPI is composed of the seven coatomer subunits and the ADP ribosylation factor (ARF) dependent GTPase. The subunits include αCOP, βCOP, β′COP, γCOP, δCOP, εCOP and ζCOP. Two protein heterodimers form the COPII complex, the Sec23p/Sec24p heterodimer and the Sec13p/Sec31p heterotetramer. COPI vesicles mediate cargo transport from the Golgi back to the ER, including escaped ER-resident proteins, while COPII mediates transport of proteins and lipids from the ER to the Golgi [21]. COPI is also required for limiting lipid storage and lipid homeostasis [22]. Immunoisolation and immunoelectron microscopy revealed that COPII were associated with poliovirus-induced cytoplasmic vesicles [23], [24], [25]. COPI activity is also required for poliovirus, EV11 and hepatitis C virus (HCV) [26], [27]. Brefeldin A (BFA), an inhibitor of COPI activity, has been shown to strongly inhibit viral RNA replication of poliovirus and EV11 [27], [28], [29]. Replication of coxsackievirus A21 (CVA21) and coxsackievirus B3 (CVB3) is also inhibited by BFA [30]. But BFA has little effect on foot-and-mouth disease virus (FMDV) and encephalomyocarditis virus (EMCV) infection and only partially repressive to Parechovirus 1 [27], [31]. Genome-wide RNA interference screening revealed that picorna-like virus, Drosophila C virus (DCV), also recruited COPI proteins to achieve viral replication [32].

In this study, using a loss of function assay based on small interfering RNA (siRNA), we were able to identify that COPI but not COPII was required for efficient EV71 replication. Loss of COPI activity protected cells from EV71 infection. Replication of EV71 was also strongly inhibited by BFA and GCA. Furthermore, we demonstrated that subunits of COPI interacted with viral protein 2C. This result suggested that COPI might be recruited to the viral replication complex through 2C, also COPI might be coupled with protein 2C to fulfill its function in virus life cycle.

## Results

### COPI but not COPII is required for EV71 replication

**Figure 1:**
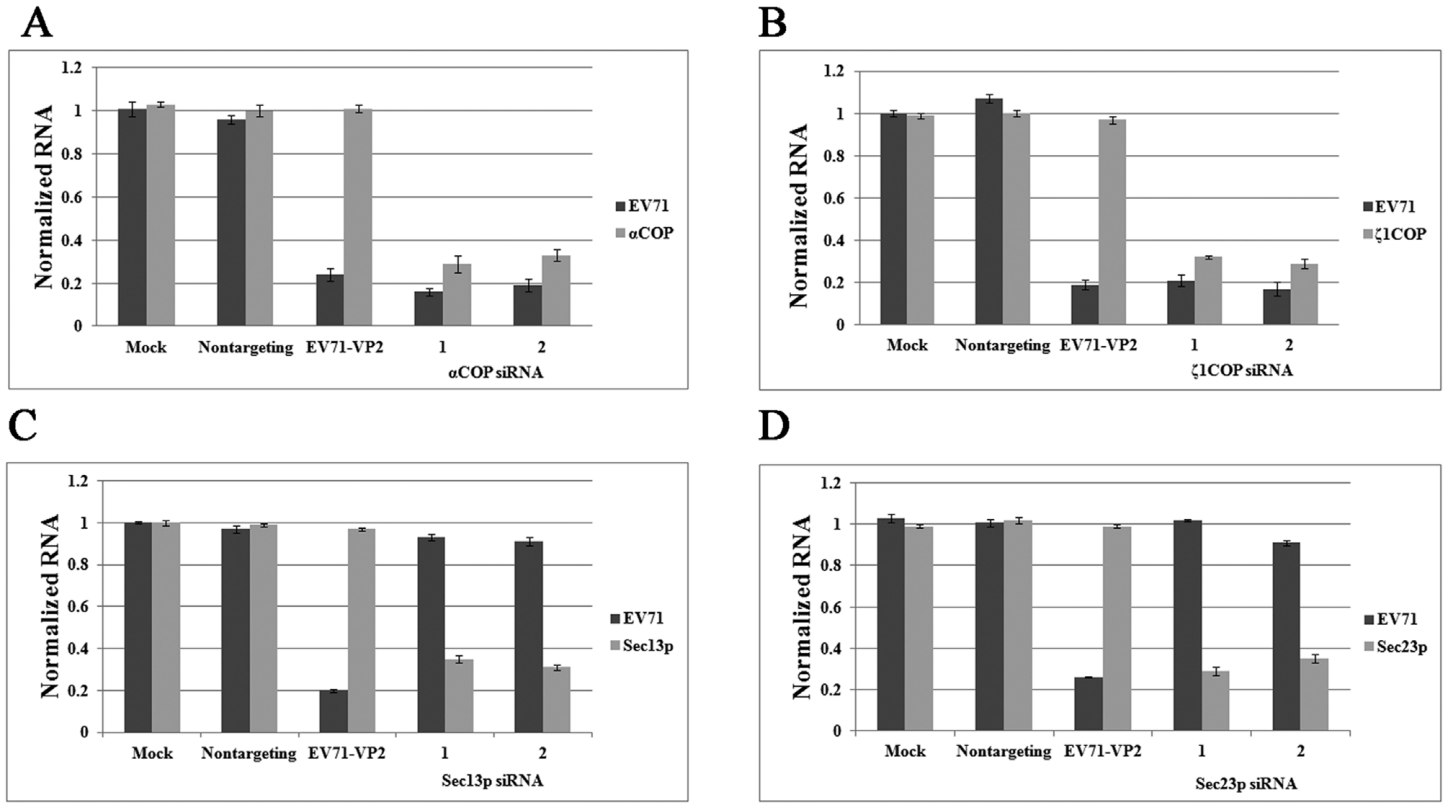
COPI but not COPII is essential for EV71 replication. COPI subunits (A) αCOP and (B) ζ1COP silencing with two individual siRNA duplexes blocked replication of EV71 (*P*<0.05). COPII subunits (C) Sec13p and (D) Sec23p depletion by siRNA was dispensable to EV71 replication (*P*>0.05). Values were obtained from triplicate wells and are mean ± SD.

We used siRNA against αCOP, ζ1COP, Sec23p, and Sec13p to block the activity of each complex. siRNA was introduced into RD cells by oligofectamine according to the manufacturer's instructions. siRNA targeting EV71 VP2 was synthesized according to the stealth-990 sequence and transfected as positive control [33]. Nontargeting siRNA was used as negative control. After 72 hours, RD cells were infected with EV71 at 0.1 multiplicity of infection (MOI). Cells were harvested and total cellular RNA was extracted at 24 hours post infection (hpi). Viral genome copies in cultured cells were determined for each group by quantitative real-time PCR. The copy number of GAPDH was used as an internal control. The copy numbers of the corresponding COPI/COPII subunits were also determined to monitor the efficacy of each siRNA. As shown in Figure 1A, compared with mock-transfected control, siRNA targeting EV71 VP2 strongly inhibited EV71 replication to around 20%, while in negative control group replication for EV71 remained unchanged. Furthermore, in contrast with mock-transfected control and negative control, both siRNA duplexes depleted αCOP mRNA transcript levels to around one third (Figure 1A). In addition, αCOP silencing strongly inhibited EV71 replication in RD cells (Figure 1A). The siRNA duplexes were almost as potent as positive control. And no significant difference was observed between the two duplexes (Figure 1A). The effect of ζ1COP silencing was also characterized. When RD cells were pretreated with siRNA against ζ1COP, transcript levels of ζ1COP and replication of EV71 were both strongly inhibited compared to the control group (Figure 1B). This was consistent with previous result, suggested that COPI was required for EV71 replication. Depletion of COPI inhibited EV71 replication in cells.

However, when the activity of COPII was interrupted by siRNA against Sec13p or Sec23p, EV71 replication remained as efficient as control group (Figure 1C, 1D). As shown in Figure 1C, in RD cells pretreated with Sec13p siRNA, the transcript levels of Sec13p were strongly inhibited, but EV71 replication was not notably altered compared with the negative control and mock control. This was similar to the results when Sec23p was knocked down by siRNA, in Figure 1D, EV71 replication was still strong. This result indicated that COPII was dispensable to EV71 replication. Overall COPI, but not COPII, was required for viral RNA synthesis in the infectious cycle.

### COPI is required for EV71 production

**Figure 2:**
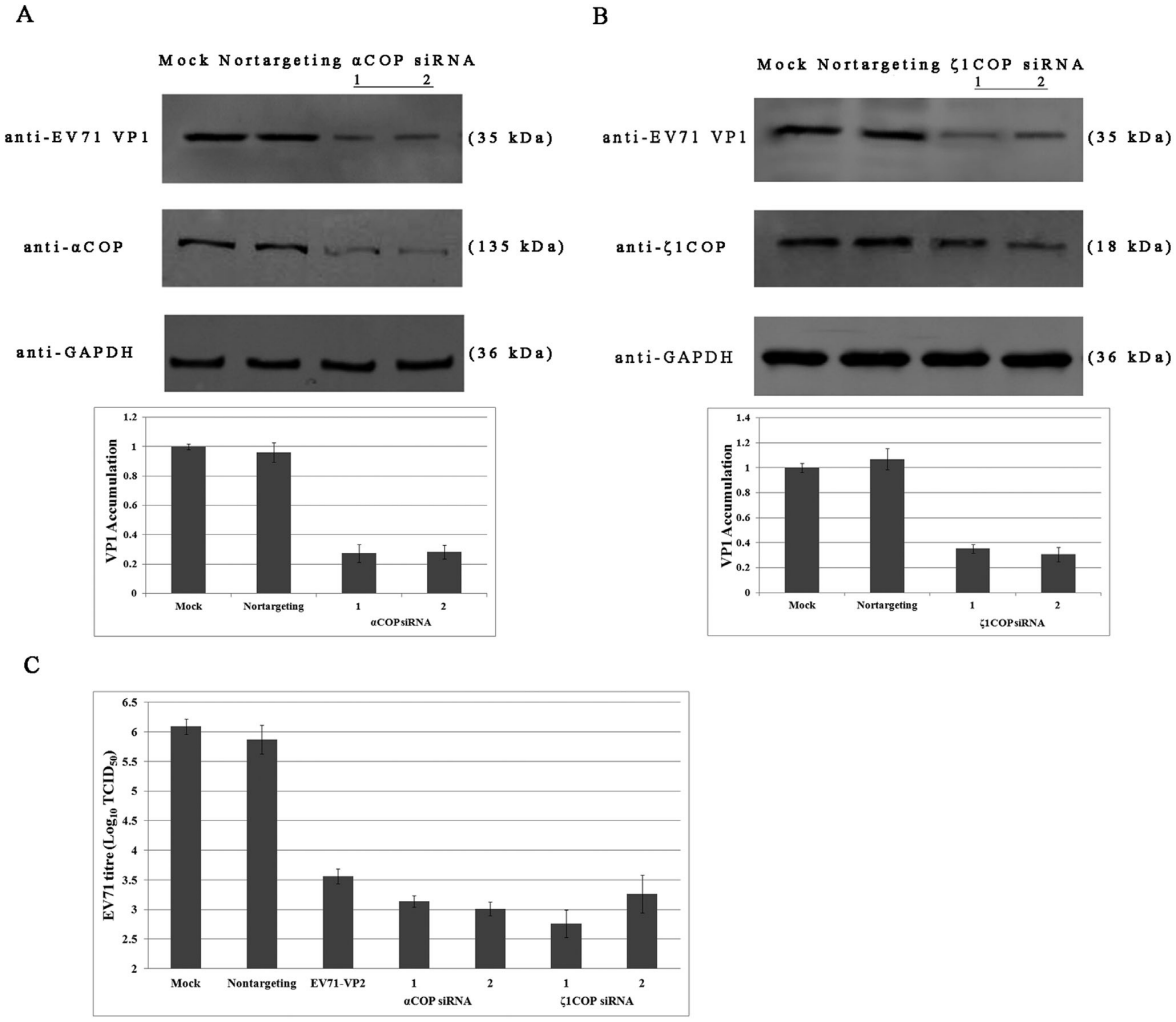
COPI is required for EV71 production. (A) αCOP, (B) ζ1COP silencing with two individual siRNA duplexes blocked EV71 viral protein VP1 expression by Western blotting with the indicated antibodies (*P*<0.05). Quantification analysis of EV71 VP1 synthesis was performed by Image J. Standard deviations of three independent experiments are shown. (C) αCOP and ζ1COP depletion by siRNA inhibited EV71 virions production (*P*<0.05). Values were obtained from triplicate wells and are mean ± SD.

To further test the dependence of viral protein synthesis and virus production on COPI for EV71, αCOP and ζ1COP siRNAs and nontargeting siRNA pretreated RD cells and mock treated RD cells, were infected with EV71 at 0.1 MOI. Cells were cultured at 37°C for an additional 24 hours. The yield of infectious EV71 from equal numbers of cells was determined. Viral protein VP1 and expression of αCOP and ζ1COP was detected by western blotting. As shown in Figure 2A, while nontargeting siRNA didn't deplete αCOP expression, αCOP was reduced to around one third by silencing siRNA duplexes compared with mock control. Western blotting assay combined with quantification analysis showed that as expression of αCOP decreased, expression of viral protein VP1 also remarkably reduced to around 30% compared with mock control and negative control (Figure 2A). When COPI activity was interrupted by ζ1COP depletion by siRNA, viral VP1 was also reduced (Figure 2B). Viral titre in the supernatants was also detected. As shown in Figure 2C, production of EV71 virions in the infectious cycle was suppressed when αCOP or ζ1COP was knocked down. A decrease of 3 to 4 orders of magnitude was observed. COPI is required for EV71 viral protein synthesis and virions production.

### COPI depletion protects RD cells from EV71 infection

**Figure 3:**
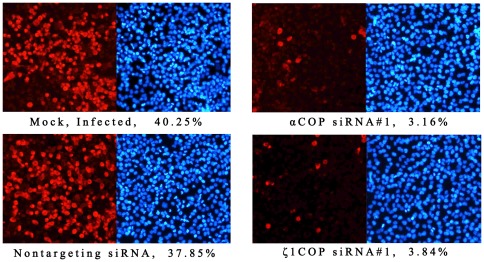
COPI depletion protects RD cells from EV71 infection. RD cells were transfected with αCOP and ζ1COP siRNA and infected with EV71. At 24 hpi, cells were fixed with 4% paraformaldehyde and permeabilized. EV71 infection was determined by immunofluorescence staining on VP1. RD cells were observed by DAPI.

To further test the effect of COPI on EV71 infection, RD cells pretreated with silencing siRNA were infected with EV71 at 0.1 MOI. Nontargeting siRNA treated and mock treated cells were infected as control. Viral infection and viral protein production was determined by immunofluorescence staining of VP1 at 24 hpi. In control group, around 40% cells were detected as red fluorescence, indicating that these cells had been infected by EV71 (Figure 3). The percentage of EV71 infected cells in nontargeting siRNA treated cells was more or less. However, in αCOP or ζ1COP depletion group, the red fluorescence was notably reduced to 3–4%, suggested that EV71 infection was inhibited (Figure 3). Silencing siRNA against COPI protected RD cells from EV71 infection.

### EV71 infection is inhibited by BFA

**Figure 4:**
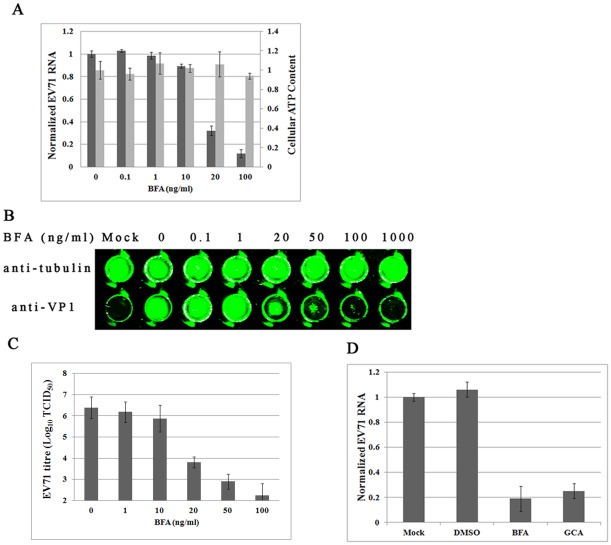
EV71 infection is inhibited by BFA. RD cells were infected with EV71 at 0.1 MOI with various concentrations of BFA. (A) Concentrations of 20 ng/ml BFA or higher significantly inhibited EV71 replication (*P*<0.05). Standard deviations of three independent experiments are shown. (B) Viral VP1 protein and internal cell tubulin protein was detected by in-cell Western blot. Concentrations of 20 ng/ml or higher BFA inhibited VP1 expression. (C) Viral titre was suppressed by 20 ng/ml or higher BFA (*P*<0.05). Standard deviations of three independent experiments are shown. (D) 50 ng/ml GCA inhibited EV71 replication in RD cells (*P*<0.05). Standard deviations of three independent experiments are shown.

To further confirm that COPI activity is required for EV71 replication, we employed the pharmacologic COPI inhibitor BFA, which blocks COPI coat assembly by inhibiting ARF-specific guanine nucleotide exchange factors. As shown in Figure 4A, BFA inhibited EV71 replication in a dose-dependent fashion, without noticeable cytotoxicity. Trace quantities of BFA, lower than 10 ng/ml, allowed marked EV71 replication. However, EV71 RNA accumulation in infected cells was reduced as the concentration of BFA increased, 20 ng/ml BFA inhibited EV71 replication effectively (Figure 4A). Replication of EV71 fell substantially to 11% compared to the negative control, when 100 ng/ml BFA was used (Figure 4A).

Accumulation of viral VP1 protein was determined by in-cell western blot. Cellular protein tubulin was used as internal control. Consistent with previous result, when BFA concentration was lower than 20 ng/ml, VP1 expression was not affected compared to control group (Figure 4B). As the concentration increased, VP1 accumulation was noticeably reduced. In addition, 50–100 ng/ml BFA markedly suppressed VP1 expression (Figure 4B). Also, expression of cellular tubulin protein was unchanged as the BFA concentration increased (Figure 4B). BFA inhibited EV71 protein accumulation in a dose-dependent fashion.

Production of infectious EV71 virions was also assayed. As expected, EV71 virion production was inhibited by BFA in a dose-dependent manner (Figure 4C). Also, a remarkable reduction was firstly observed when BFA concentration reached 20 ng/ml and a decrease of 4 orders of magnitude was observed when 100 ng/ml BFA was added (Figure 4C). Taken together EV71 infection was inhibited by BFA in cells.

Similar to BFA, Golgicide A (GCA) is also an ARF-specific guanine nucleotide exchange factor inhibitor to inhibit COPI activity. To test whether EV71 replication could be inhibited by GCA, 50 ng/ml GCA was added into the culture medium at the time of infection. DMSO and 50 ng/ml BFA was used as negative and positive control. Viral genome copies were monitored by quantitative real-time PCR 24 hpi. As shown in Figure 4D, GCA inhibited EV71 replication to a similar extent as BFA.

### COPI is required downstream of viral entry

**Figure 5:**
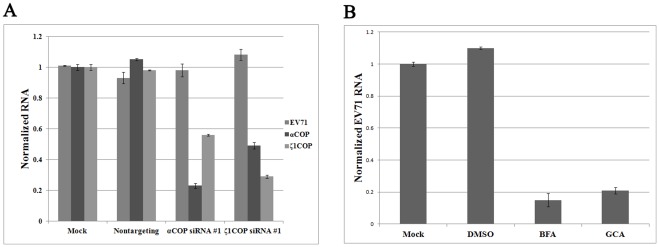
COPI is required downstream of viral entry. (**A**) Cells were pretreated with siRNA, infected at 4°C to allow surface binding, followed by 3 hr at 25°C to resume endocytosis. Viral entry was measured by determining viral genome copies, which was almost unchanged between COPI knocked down group and control group (*P*>0.05). (**B**) BFA and GCA inhibited EV71 replication (*P*<0.05), when added up to 6 hpi. RD cells were infected with at 0.1 MOI and harvested at 24 hpi. The experiment was performed in triplicate, and the bars represent means ± SD.

To determine whether depletion of COPI by siRNA treatment affect EV71 entry into RD cells, viral genome copies for virions during entry through endocytosis were monitored. As shown in Figure 5A, in nontargeting siRNA pretreated RD cells, viral genome copy numbers were unaltered compared with mock treated group. Meanwhile, viral genome copies remained as many as control group in αCOP and ζ1COP siRNA pretreated RD cells, although copy numbers for αCOP and ζ1COP fell to around 30% (Figure 5A). This result indicated that depletion of COPI didn't reduce EV71 entry into RD cells. Furthermore, 100 ng/ml BFA and GCA inhibited EV71 replication when added up to 6 hpi in Figure 5B, also suggesting COPI activity was required for a step in the EV71 lifecycle postentry. Overall COPI is required downstream of viral entry.

### COPI interact with viral protein 2C

**Figure 6:**
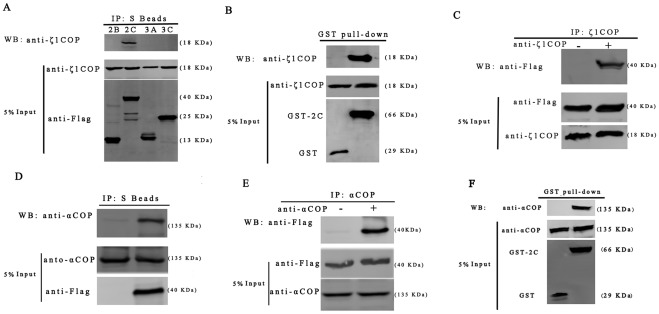
COPI interact with viral protein 2C. (A) At 24 hpt, immunoprecipitation was conducted with S beads and western blotting was carried out as indicated. (B) GST pull-down was performed with 293T cell lysates. Western blotting detection was carried with indicated antibodies. (C) Immunoprecipitation was conducted with Protein A agarose plus anti-ζ1COP antibody and western blotting was carried out as indicated. (D) Immunoprecipitation was conducted with S beads and western blotting was carried out as indicated. (E) Immunoprecipitation was conducted with Protein A agarose plus anti-αCOP antibody and western blotting was carried out as indicated.

To investigate interaction between COPI subunits and EV71 viral proteins, co-immunoprecipitation was performed. S-flag-tag labeled viral nonstructural proteins, 2B, 2C, 3A and 3C, were over-expressed in 293T cells. Co-immunoprecipitation was performed by the S tag on the N-terminus according to the manufacture's procedure. As shown in Figure 6A, all four nonstructural viral proteins were expressed at high level in cells indicated by the flag tag detection. However, ζ1COP could be only captured when co-immunoprecipitated with EV71 2C protein, suggesting interaction between COPI and viral 2C protein, but not 2B, 3A and 3C. The interaction was further confirmed by GST pull-down assay. As shown in Figure 6B, ζ1COP was captured by GST-2C, but not GST alone, indicating that ζ1COP bound viral 2C protein in vitro. The interaction between ζ1COP and 2C was further confirmed by pulling down ζ1COP and testing viral 2C. As shown in Figure 6C, flag-tagged 2C was captured by anti-ζ1COP. Next, interaction between αCOP and viral 2C was tested. In Figure 6D and 6E, αCOP was also captured by viral 2C and vice versa. Taken together, these results suggested interaction between COPI and viral 2C protein.

## Discussion

Genomic siRNA screens can identify roles for genes and biological pathways in viral infection by their function. This technology has been successfully applied to the identification of novel host dependency factors for HIV, HCV and DCV [26], [34], [35], [36]. In this study, we designed duplexes of siRNA against COPI and COPII proteins, then conducted siRNA a loss-of-function assay. We demonstrated that EV71 viral RNA replication is dependent on COPI, but not COPII. The finding that the COPI inhibitor, BFA and GCA, strongly inhibited EV71 replication further confirmed the dependence. The COPI proteins interacted with viral protein 2C by co-immunoprecipitation assay and GST pull-down assay.

Picornaviruses induce the formation of a cytoplasmic vesicular compartment in infected cells and that this compartment is the essential site of viral RNA replication. Previous studies showed that membrane derived from the ER via a COPII coatomer-mediated process or the Golgi via COPI was constantly associated with picornavirus replication [24], [27]. Consistent with previous results, our result suggested that the replication of EV71 was dependent on COPI. However, compared with poliovirus dependence on COPII, COPII seemed to be dispensable to EV71. This might because of the limitation of our siRNA screen assay, or because of the preference of EV71 for COPI.

BFA is a fungal metabolite used to study membrane traffic in eukaryotic cells. BFA blocks protein transport from the ER to the Golgi apparatus [37], [38]. BFA prevents membrane binding of COPI and formation of COPI-coated vesicles [39], [40], while the formation of COPII-coated vesicles is not affected [41], [42]. BFA also prevents budding of clathrin-γ-adaptin-coated vesicles from the Golgi apparatus, endosomes, and lysosomes [43], [44]. However, replication of poliovirus was not affected in cells in which clathrin-γ-adaptin association with membranes was inhibited [45]. Similar to BFA, GCA was also shown to inhibit ARF inactivation and to block COPI activity. Both BFA and GCA strongly inhibited EV71 replication. Thus, our results, in combination with the published data, strongly suggest the inhibitory effect of BFA is due to prevention of COPI activity.

In addition to structural proteins, picornaviruses produce several precursor polyproteins and mature protein products. Because of the limited coding capacity of picornavirus genomes, both the precursors and mature products actively participate in viral processes and are involved in the assembly of replication initiation complexes and mediate the process of RNA synthesis. In this study, we conducted a co-immunoprecipitation assay with four viral proteins: 2B, 2C and 3A, 3C. αCOP and ζ1COP was captured by 2C, suggesting interaction between 2C and COPI. GST pull-down assay confirmed the interaction. Together with poliovirus, 2C induces intracellular membrane rearrangement and the formation of viral-induced cytoplasmic vesicles [46], [47], a poliovirus mutant carrying mutations on 2C protein was resistant to BFA [48], this result indicated EV71 2C protein could be involved in the recruitment of COPI into the replication complex.

Picornaviruses usually disrupt the secretory pathway in infected cells, which could include a block in the secretion of interferons and proinflammatory cytokines or the inhibition of cell surface MHC class 1 expression and antigen presentation. Poliovirus 3A, 2B, 2C and 2BC proteins are identified as involved in this process [49], [50], [51]. The 3A protein of coxsackievirus B3 (CVB3) and poliovirus was shown to interact with GBF1, a guanine nucleotide exchange factor for ARF, and block COPI assembly [52], [53]. Mutations in 3A rescue growth of poliovirus and CVB3 in the presence of BFA [54], [55]. For foot-and-mouth disease virus (FMDV), viral proteins 2B, 2C and 2BC block endoplasmic reticulum-to-Golgi transport [56], [57]. Similar roles for EV71 have not yet been identified. Our finding that EV71 2C protein could interact with COPI is consistent with previous result that FMDV 2C protein could colocalize with ζ1COP to the Golgi apparatus, and arrest transport within the Golgi apparatus [57], which might suggest that EV71 2C functions the same way as FMDV 2C in disruption of the secretory pathway in infected cells. However, BFA has little effect on FMDV replication suggesting FMDV may acquire cellular membranes into its replication complexes in a manner different from that of EV71, while the block of secretion by 2C has the potential to contribute to the development of persistent infections rather than viral RNA replication [31].

Finally, the fact that inhibition of COPI could protect cells from EV71 infection, suggests a novel approach to viral inhibition. Since no effective therapy is available for the treatment of EV71 infection, modulating host COPI activity could be a promising new direction for antiviral drug discovery.

## Materials and Methods

### Cell culture and drug treatment

Rhabdomyosarcoma (RD, ATCC, USA) cells and HEK293T (293T, ATCC, USA) cells were propagated and maintained in Minimum Essential Medium (MEM, HyClone, Logan, USA) and Dulbecco's Modified Eagle Medium (DMEM, HyClone, Logan, USA) supplemented with 10% fetal bovine serum (FBS) (Invitrogen, CA, USA) and 100 U/ml penicillin, and 100 μg/ml streptomycin at 37°C with 5% CO_2_. Brefeldin A (BFA, Sigma-Aldrich, St. Louis, USA) was dissolved in ethanol and stored at 1 mg/ml in 4°C before use. Golgicide A (GCA, Sigma-Aldrich, St. Louis, USA) was dissolved in dimethyl sulfoxide (DMSO) and stored at 1 mg/ml. BFA and GCA were added to cell cultures in a concentration series at the time of infection or indicated time.

### Virus infection

EV71 strain (Shzh-98, GenBank accession no. AF302996) was used. Viruses were propagated in RD cells and infected at 0.1 multiplicity of infection (MOI) per cell, measured as 50% tissue culture infectious doses (TCID_50_).

### Plasmid construction

For transient expression in 293T cell, EV71 2B, 2C and 3A, 3C were cloned from EV71 Shzh-98 strain and constructed into pcDNA3 vector (Invitrogen, CA, USA) under the control of CMV promoter. A S-protein tag (KETAAAKFERQHMDS) and a Flag tag (DYKDDDDK) were tandem anchored on the N- terminus of these proteins for co-immunoprecipitation and western blotting. For expression and purification of GST-fused 2C protein, EV71 2C was constructed in the pGEX-6P-1 vector (GE Healthcare, NJ, England) to generate pGEX-6P-2C. The nucleotide sequences of the plasmids and the orientation of the inserted fragments were verified by sequencing.

### siRNA design and transfection

The siRNAs were designed using a Web-based siRNA tool BLOCK-iTTM RNAi Designer (Invitrogen, https://rnaidesigner.invitrogen.com/rnaiexpress/) and were custom synthesized by Invitrogen (CA, USA). Sequences are in Table 1. siRNA targeting the EV71 VP2 protein was synthesized according to the Stealth-990 sequence and used as a positive control [33]. Stealth RNAi siRNA Negative Control Med GC (Invitrogen, CA, USA) was used as a negative control. siRNA was introduced into RD cells by transfection using Oligofectamine Reagent (Invitrogen, CA, USA) according to the manufacturer's instructions. RD cells were cultured overnight until 40% confluence before use; 100 pmol siRNA was incubated in 60 μl Opti-MEM® (Invitrogen, CA, USA) for 15 min at room temperature (RT), while 5 μl Oligofectamine was incubated with 15 μl Opti-MEM. The siRNA and Oligofectamine were mixed and incubated for 20 min at RT before addition to cell cultures. The culture medium was changed 4 hr later and cells were cultured 72 hr before virus infection.

**Table 1:** siRNA sequences used in this study.

Target Gene	Duplex	Sense Sequence
αCOP	1	5′-GGCGCAUGAAUGAAUCAAATT-3′
	2	5′-GGCCAUUACAACAAUGUAUTT-3′
ζ1COP	1	5′-CCAAAGAACAGAUCAAGUGTT-3′
	2	5′-GGCUGUGGAUGAAAUUGUATT-3′
Sec13p	1	5′- CUUUGAUGUGCGCAAUGGATT-3′
	2	5′-GAAGAUCAACAACGCUCACTT-3′
Sec23p	1	5′-UAUGAACCUGUGCUUUGCATT-3′
	2	5′GAUCUCCAUUUCUUCAAGUTT-3′
Stealth-990	5′-GCGCAAUUAACUAUUGGCAACUCCA-3′	

* Stealth-990 corresponds to the siRNA against EV71 VP2.

### Quantitative real-time PCR

Total cellular RNA and viral RNA were extracted from each well using RNAeasy Mini kit (Qiagen, Hilden, Germany) at 24 hpi according to the manufacturer's instructions. Reverse transcribed PCR was conducted using Superscript First-Strand Synthesis System (Invitrogen, CA, USA) in 20 μl with 1.2 μg total RNA according to the manufacturer's protocol. Relative quantitative real-time PCR was conducted on an ABI Prism 7000 Real-time PCR System (Applied Biosystems, CA, USA) by using a Power SYBR Green PCR Master Kit (Applied Biosystems, CA, USA). Reactions were 2 μl cDNA, 1 μl of each primer and 25 μl Power SYBR Green PCR Master Mix in 50 μl. Efficiency-corrected relative quantitation was used with GAPDH as an internal control [58]. Primer sequences are in Table 2.

**Table 2:** Quantitative real-time PCR primers.

Target	Forward Primer	Reverse Primer
EV71	CCCCTGAATGCGGCTAAT	CAATTGTCACCATAAGCAGCCA
αCOP	CCTGGGATGAGAGTGGGGTAT	AGTGCTTTGGCTGCTTCC
ζ1COP	AAGATGGAGGCGCTGATTTT	ACACTGGGGTAGGTGTCGTC
Sec13p	ACGAGCATGCGGGACACGACT	TGGGAGCAGCTGGCGATGGT
Sec23p	TGTGCCCTTGATCAAACTGGA CTT	GATAATGCGTGACAAACTGGAT GG
GAPDH	CTCTGCTCCTCCTGTTCGAC	TTAAAAGCAGCCCTGGTGAC

### Western blotting

RD cells were collected 24 hpi and washed with PBS twice and lysed in buffer containing 100 mM NaCl, 20 mM Tris (pH 8.0), 0.5% NP-40, 0.25% sodium deoxycholate, 1 mM EDTA with proteinase inhibitor cocktail. Supernatant was collected after centrifuged at 13,000 rpm/min for 15 min and separated by electrophoresis in denaturing 4 to 10% SDS-PAGE and transferred to nylon polyvinylidene difluoride (PVDF) membranes (Hybond P, Piscataway, USA). Membranes were blocked with 5% nonfat dry milk and probed with primary antibodies as indicated at 4°C overnight, followed by incubation with the corresponding IRD Fluor 680-labeled IgG or IRD Fluor 800-labeled IgG secondary antibody (Li-Cor Inc., NE, USA). After washing, membranes were scanned using an Odyssey Infrared Imaging System (Li-Cor, NE, USA) at the recommended wavelength and analyzed with Odyssey software. Molecular sizes of proteins were determined by comparison with prestained protein markers (Fermentas, Maryland, USA). EV71 capsid protein VP1 was detected by anti-EV71 VP1 monoclonal antibody (eENZYME, Maryland, USA). αCOP and ζ1COP were detected by rabbit anti-αCOP and anti-ζ1COP antibody (ABcam, Cambridge, England). GST or GST-fused 2C protein were identified with mouse anti-GST (Beyotime, Suzhou, China) and corresponding secondary antibody. To control for protein loading, levels of housekeeping protein GAPDH were assessed using mouse anti-GAPDH (Beyotime, Suzhou, China) and IRD Fluor 680-labeled IgG secondary antibody (Li-Cor Inc., NE, USA). Quantification analysis of EV71 VP1 synthesis in western blot was performed by Image J.

### Immunofluorescence staining

RD cells were pretreated with silencing siRNA or negative control siRNA for 72 hr and infected with EV71 at 0.1 MOI. At 24 hpi, cells were fixed with 4% paraformaldehyde for 30 min and permeabilized with 0.5% Triton X-100 for 30 min at RT. Samples were washed twice with PBS and blocked with 5% goat serum and incubated with mouse anti-EV71 VP1 (eENZYME, Maryland, USA) for 60 min. After washed twice with PBS, samples were incubated with Alexa Fluor 568 rabbit anti-mouse IgG (Invitrogen, CA, USA) for another 30 min. Cells were counterstained with DAPI to visualize nuclear DNA.

### In-cell Western blot analysis

In-cell Western blot analyses were performed as described previously [59]. siRNA pre-treated RD cells were cultured at 37°C with 5% CO_2_ for 72 hr and infected with EV71 at 0.1 MOI. At 24 hpi, cells were fixed with 4% paraformaldehyde for 30 min and permeabilized with 0.5% Triton X-100 for 15 min at RT. Cells were washed twice with PBS and incubated with anti-EV71 VP1 monoclonal antibody (eENZYME, Maryland, USA) and mouse anti-tubulin (Beyotime, Suzhou, China) overnight at 4°C. The next day, cells were washed with 0.1% Tween-20 in PBS and incubated with goat anti-mouse 680 (1∶500) (Li-Cor, NE, USA). After washing twice with PBST and twice with PBS, cells were scanned using an Odyssey Infrared Imager (Li-Cor Inc., NE, USA).

### Viral titre assay

Virus titre in supernatants was determined as TCID_50_ on RD cells by the Reed-Muench method [60]. Results were obtained in three independent experiments and the representative results are shown.

### Viral entry assay

Cells were pretreated with siRNA for 3 days and placed at 4°C to block endocytosis. Virions were added at 5 MOI and incubated for 2 h to be allowed to bind to the surface of RD cells. The cells were washed twice with PBS to remove unbound virions and then incubated at room temperature to resume endocytosis. Three hours later, total cellular RNA and viral RNA was extracted and determined by quantitative real-time PCR to monitor virions during entry.

### Transfection and Co-immunoprecipitation assay

Cell transfection was performed using Lipofectamine 2000 (Invitrogen, CA, USA) following the manufacturer's protocol. Briefly, 10 μg of plasmids were mixed with 30 μl Lipofectamine 2000 and incubated at RT for 20 min before added into cell culture. Transfected 293T cells were cultured for another 24 hr. Cells were collected and lysed in buffer containing 100 mM NaCl, 20 mM Tris (pH 8.0), 0.5% NP-40, 0.25% sodium deoxycholate, 1 mM EDTA with proteinase inhibitor cocktail. The supernatants were cleared at 14,000 rpm to remove debris and incubated with S protein beads (Novagen, Darmstadt, Germany) overnight at 4°C. The immunocomplexes were recovered by a brief centrifugation and washed three times with PBS and subjected to SDS-PAGE. Protein bands were detected by western blotting. Reciprocal co-immunoprecipitation assay was conducted by Protein A agarose (Invitrogen, CA, USA) plus 2 μg Goat anti-αCOP/anti-ζ1COP antibody (ABcam, Cambridge, England) with cell protein extract overnight at 4°C. The immunocomplexes were recovered by a brief centrifugation and washed three times then subjected to SDS-PAGE for western blotting detection with anti-flag antibody.

### GST pull-down assay

To further confirm the interaction between COPI complex and EV71 2C protein, a GST pull-down assay was assessed. Total protein was extracted from about 5×10^7^ 293T cells in buffer containing 100 mM NaCl, 20 mM Tris (pH 8.0), 0.5% NP-40, 0.25% sodium deoxycholate, 1 mM EDTA with proteinase inhibitor cocktail. Cell debris was removed by centrifugation. Recombinant GST-2C and GST alone were expressed in *Escherchia coli* BL21 strain and purified using glutathione beads according to standard protocols. GST–2C and GST alone was incubated with 293T cell protein extract at 4°C for 2 hr. Glutathione beads were recovered by a brief centrifugation and washed three times with PBS before resolved by SDS-PAGE and detected by western blotting.
